# TJ-M2010-5, a novel CNS drug candidate, attenuates acute cerebral ischemia-reperfusion injury through the MyD88/NF-κB and ERK pathway

**DOI:** 10.3389/fphar.2022.1080438

**Published:** 2022-12-15

**Authors:** Zeyang Li, Minghui Zhao, Xiaoqian Zhang, Yiran Lu, Yang Yang, Yalong Xie, Zhimiao Zou, Liang Zhou, Runshi Shang, Limin Zhang, Fengchao Jiang, Dunfeng Du, Ping Zhou

**Affiliations:** ^1^ Institute of Organ Transplantation, Tongji Hospital, Tongji Medical College, Huazhong University of Science and Technology, Wuhan, China; ^2^ Key Laboratory of Organ Transplantation, Ministry of Education, NHC Key Laboratory of Organ Transplantation, Key Laboratory of Organ Transplantation, Chinese Academy of Medical Sciences, Wuhan, China; ^3^ Department of Neurology, Union Hospital, Tongji Medical College, Huazhong University of Science and Technology, Wuhan, China; ^4^ Wuhan Yangtze International School, Wuhan International Educational Center, Wuhan, China; ^5^ Academy of Pharmacy, Tongji Medical College, Huazhong University of Science and Technology, Wuhan, China

**Keywords:** TJ-M2010-5, drug, cerebral ischemia-reperfusion injury, neuroinflammation, Myd88 inhibitor

## Abstract

**Background:** Cerebral ischemia-reperfusion injury (CIRI) inevitably occurs after vascular recanalization treatment for ischemic stroke. The accompanying inflammatory cascades have a major impact on outcome and regeneration after ischemic stroke. Evidences have demonstrated that TLR/MyD88/NF-κB signaling contributes to CIRI. This study aimed to investigate the druggability of MyD88 in the central nervous system (CNS) and the neuroprotective and anti-neuroinflammatory effects of the MyD88 inhibitor TJ-M2010-5 on CIRI.

**Methods:** A middle cerebral artery occlusion (MCAO) model was used to simulate CIRI in mice. BV-2 cells were stimulated with oxygen glucose deprivation/reoxygenation (OGD/R) or lipopolysaccharide, and SH-SY5Y cells were induced by OGD/R *in vitro*. Neurological deficit scores and cerebral infarction volumes were evaluated. Immunofluorescence staining was performed to measure neuronal damage and apoptosis in the brain. The anti-neuroinflammatory effect of TJ-M2010-5 was evaluated by analyzing the expression of inflammatory cytokines, activation of microglia, and infiltration of peripheral myeloid cells. The expression of proteins of the MyD88/NF-κB and ERK pathway was detected by Simple Western. The concentrations of TJ-M2010-5 in the blood and brain were analyzed by liquid chromatography-mass spectrometry.

**Results:** The cerebral infarction volume decreased in mice treated with TJ-M2010-5, with the most prominent decrease being approximately 80% of the original infarction volume. Neuronal loss and apoptosis were reduced following TJ-M2010-5 treatment. TJ-M2010-5 inhibited the infiltration of peripheral myeloid cells and the activation of microglia. TJ-M2010-5 also downregulated the expression of inflammatory cytokines and inhibited the MyD88/NF-κB and ERK pathway. Furthermore, TJ-M2010-5 showed good blood-brain barrier permeability and no neurotoxicity.

**Conclusion:** TJ-M2010-5 has an excellent therapeutic effect on CIRI as a novel CNS drug candidate by inhibiting excessive neuroinflammatory responses.

## Introduction

Stroke is an acute cerebrovascular disease in which focal neurological loss suddenly occurs in the relevant parts of the brain due to infarction or hemorrhage (Hankey, 2017). The most common type of stroke is ischemic stroke, accounting for 70%–80%. The cornerstone of effective ischemic stroke care continues to be timely reperfusion treatment, either intravenous recombinant tissue plasminogen activator (rtPA) and/or mechanical thrombectomy (Prabhakaran et al., 2015). Although clinical use of intravenous rtPA and/or mechanical thrombectomy result in high reperfusion rates of acute cerebral infarction, the benefits of reperfusion therapy are incomplete in about half of the patients treated (Chamorro et al., 2021; van Horn et al., 2021). When reperfusion occurs, a seemingly paradoxical increased injury can occur, such as hemorrhagic transformation, which limits the use of rtPA (Liu et al., 2017). Evidence has shown that acute immune-inflammatory reactions related to reperfusion can lead to secondary brain injury and expand the scope of brain injury (Iadecola and Anrather, 2011; He et al., 2021; Przykaza, 2021). Cerebral ischemia-reperfusion injury (CIRI) inevitably occurs after cerebral infarction. CIRI makes the original ischemic necrosis area more than double, and a specific drug is lacking in clinical practice. Studies have shown that myeloid differentiation factor 88 (MyD88) plays a vital role in CIRI (Wang et al., 2011; Ma et al., 2020; Zhong et al., 2020; Qin et al., 2022).

Following ischemic stroke, damaged neurons release damage associated molecular patterns (DAMPs), such as high mobility group box 1 (HMGB1) (Singh et al., 2016). DAMPs spread when reperfusion and were sensed by toll-like receptor (TLR), leading to a series of inflammatory cascade (Akira and Takeda, 2004; Hanisch et al., 2008). MyD88 is an adaptor molecule linking TLR or interleukin (IL) receptors signaling to the downstream activation of nuclear factor-κB (NF-κB) (Janssens and Beyaert, 2002). TLRs identify DAMPs and activate MyD88/NF-κB and ERK signaling, resulting in the expression of pro-inflammatory factors (Li et al., 2018; Chen et al., 2020). Furthermore, inflammatory factors enhance immune cell activation and regulate the cell death of inflammatory tissues (Takeuchi and Akira, 2010), which increases DAMPs, leading to the inflammatory cascade and expanding the scope of damage (Silvis et al., 2020). Blocking the TLR/MyD88/NF-κB signaling can downregulate inflammatory responses and alleviate CIRI (Gao et al., 2009; Bohacek et al., 2012; Wang et al., 2016).

**FIGURE 1:**
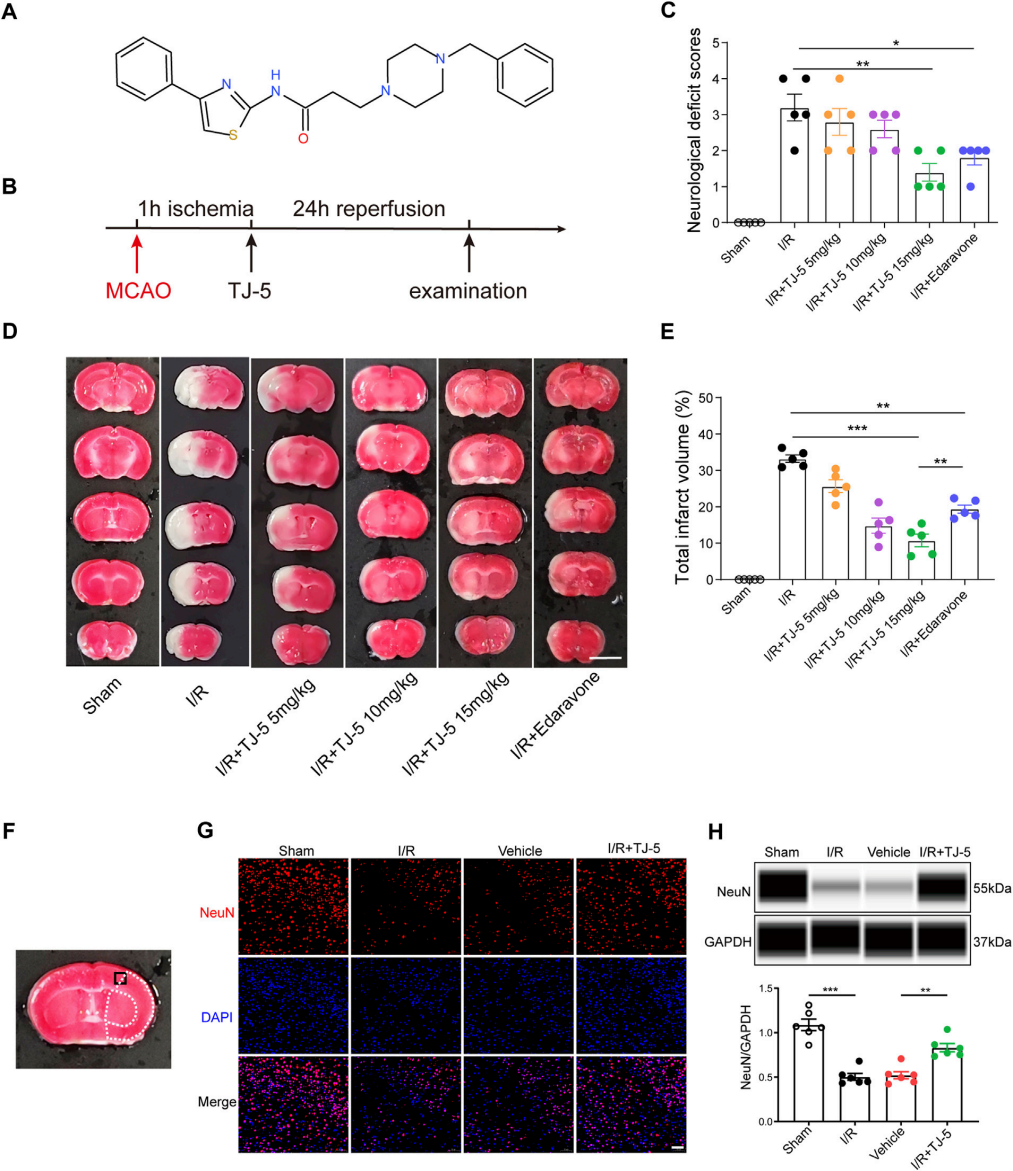
Neuroprotective effect of TJ-5 treatment in CIRI mice. **(A)** The molecular structure of TJ-5. **(B)** Experimental timeline of TJ-5 treatment in CIRI mice. **(C–E)** Representative TTC-stained slices at 24 h after reperfusion (scale bar = 5 mm) and statistical analysis of neurological deficit score and infarct volume. Values are mean ± SEM and analyzed by two-way ANOVA. (*p < 0.05, **p < 0.01, ***p < 0.001). **(F)** Black boxed area illustrates cortical region represented in the NeuN, TUNEL and Iba-1 images. White dashed line areas illustrate ischemic core (medial) and peri lesion (lateral) regions. **(G)** Representative NeuN immunofluorescence staining image (scale bar = 50 μm). **(H)** NeuN protein levels in the brain were analyzed. All the experiments were repeated three times. Values are mean ± SEM. (**p < 0.01, ***p < 0.001).

By analyzing the structural domain of MyD88 and using computer-aided systems such as drug design and virtual screening, the small-molecule aminothiazole derivative MyD88 inhibitor, TJ-M2010 series (WIPO Patent Application Number: PCT/CN 2012/070811) has been innovatively developed, which can specifically bind to the Toll/Interleukin-1 receptor (TIR) domain of MyD88 and prevent homodimerization of MyD88. TJ-M2010-5 (TJ-5), one of TJ-M2010 series, has the best water solubility and bioavailability. The chemical structure of TJ-5 (Figure 1A) and its interaction with the MyD88 TIR domain have been described in a previous study (Xie et al., 2016). Specifically, TJ-5 interacts with amino-acid residues of αE, βD, βC, αA, DD loop, and EE loop of MyD88 with a nonbond interaction and the energies (docking score) is -883.298 kJ/mol (Xie et al., 2016). Our previous studies have shown that TJ-5 can inhibit the activation of peripheral innate immune cells such as macrophages and dendritic cells in hepatic, myocardial, and renal ischemia-reperfusion (I/R) animal models (Zhang et al., 2016; Miao et al., 2020; Zhou et al., 2022). However, the druggability of MyD88 is still unknown, especially in central nervous system (CNS) diseases. Due to the requirement of CNS drugs to cross the blood-brain barrier (BBB) and the frangibility of neurons to ischemia and hypoxia, as well as the particularity of the central nervous immune system (Banks, 2016), the effect and mechanism of MyD88 inhibition against CIRI are not clear. Here, we focused on the anti-neuroinflammatory effect of TJ-5. The neuroprotective potential of TJ-5 as a CNS drug candidate for CIRI treatment was evaluated.

## Materials and methods

### Animals and groups

Male C57BL/6 mice (Beijing Vital River Laboratory Animal Technology Co. Ltd., Beijing, China) weighing 22–28 g and aged 8–10 weeks were used. All animal experiments were approved by the Institutional Animal Care and Use Committee of Tongji Hospital (Wuhan, China). All procedures were performed in accordance with specific pathogen-free standards. Mice were randomly divided into the following seven groups: sham, I/R, vehicle (I/R + saline), I/R + TJ-5 (5 mg/kg), I/R + TJ-5 (10 mg/kg), I/R + TJ-5 (15 mg/kg), and I/R + edaravone (3 mg/kg).

### Cerebral ischemia-reperfusion injury model

The mice were anesthetized with 1% pentobarbital sodium solution *via* intraperitoneal injection, and the body temperature was maintained at 37.0°C–37.5°C. The middle cerebral artery occlusion (MCAO) model was established according to the Longa method, as described previously (Longa et al., 1989). In brief, the right carotid artery was carefully separated and exposed. A silicon-coated embolic suture (Doccol, United States) was inserted slowly from the external carotid artery (ECA) into the internal carotid artery (ICA) until it reached the middle cerebral artery (MCA). After 1 h of ischemia, the embolic suture was withdrawn. The surgical procedure in the sham group was the same as that in the I/R group, but the MCA was not obstructed.

### Neurological deficiency score

After 24 h of reperfusion, each group of mice was scored blindly, according to the scoring system of the Longa method (Longa et al., 1989). The score was 0 for no obvious neurological deficit, one for inability to fully extend the left forelimb, two for turning to the left, three for leaning to the left while walking, and four for the inability to walk spontaneously and impaired consciousness.

### TTC staining

The mice were sacrificed 24 h after I/R. Mouse brains were harvested for the measurement of cerebral infarct volume. The brains were frozen at −80°C for 5 min and cut into five 2-mm thick slices. The slices were then placed in a small dish, 2% 2,3,5-triphenyltetrazolium chloride (TTC) solution (Sigma, United States) was added, and 4% paraformaldehyde was used for fixation. Images were captured using a digital camera. ImageJ software was used to measure the volume of the brain infarction.

### Cell culture and treatment

BV-2 microglial and SH-SY5Y cells were cultured as previously described (Zhao et al., 2020). A lipopolysaccharide (LPS)-stimulated BV-2 cell model was established to mimic severe neuroinflammation induced by multiple inflammatory mediators after reperfusion (Li et al., 2021). BV-2 cells were pretreated with different concentrations of TJ-5 for 2 h before LPS (1 μg/ml) stimulation (#L2880, Sigma, United States). Twenty-4 hours later, BV-2 cells and the culture supernatants were harvested for subsequent experiments. In addition, ST2825, a recognized MyD88 inhibitor, was used as positive control group.

The oxygen glucose deprivation/reoxygenation (OGD/R)-induced SH-SY5Y cell model was established to mimic CIRI as previously described (Dong et al., 2021). Briefly, SH-SY5Y cells were cultured in glucose-free Dulbecco’s Modified Eagle Medium, and the flasks were placed inside an incubator (1% O_2_, 94% N_2_, 5% CO_2_) for 4 h. OGD cells were then incubated in standard culture conditions with or without TJ-5 for 24 h of reperfusion. OGD 4 h/R 24 h SH-SY5Y cells were harvested, and the Annexin V/PI Apoptosis Kit (Multi- Science Biotech, China) was used to detect apoptosis by flow cytometry.

### CCK-8 assay

BV-2 and SH-SY5Y cells were seeded in a 96-well plate at a density of 1 × 10^4^ cells/well. Twenty-4 hours later, different concentrations of TJ-5 (0, 1, 5, 10, 20, and 30 μM) were added and co-cultured with cells for 24 h. Cell Counting Kit-8 (CCK8, Sigma, Shanghai, China) was used to measure cell viability. Experiments were conducted according to the manufacturer’s instructions. After incubation at 37°C for 2 h, absorbance was measured at 450 nm using a microplate reader (Synergy 2, BioTek Instruments, United States).

### Immunofluorescence staining

As mentioned previously, immunofluorescence staining was performed on paraffin-embedded brain slices (Ye et al., 2020). After standard histological procedures, the slices were treated with the TUNEL (terminal deoxynucleotidyl transferase dUTP nick end labeling) reaction mixture to detect apoptosis according to the manufacturer’s protocol (Roche, Germany). Furthermore, the slices were used for immunofluorescence with rabbit anti-NeuN antibody (1:300, CST) and rabbit anti-Iba-1 antibody (1:500, CST). After incubating overnight at 4°C, goat anti-rabbit IgG (1:1000) secondary antibodies were applied, and the brain slices were incubated for 2 h at room temperature. Finally, the slices were washed and labeled with 4′,6-diamidino-2-phenylindole for 10 min at room temperature. Images were captured using a fluorescence microscope.

For BV-2 cells immunofluorescence staining, the cells were fixed with 4% paraformaldehyde for 10 min and permeabilized with 0.5% Triton X-100 for 10 min. After blocking with goat serum, the cells were sequentially incubated with the primary antibody, secondary antibody, and Hoechst in sequence. Images were captured using a confocal microscope. The fluorescence intensity was analyzed using ImageJ software.

### Quantitative real-time PCR

Total mRNA was extracted from the brain cortex using TRIzol reagent and reverse-transcribed into cDNA using the PrimeScript™ RT reagent kit (Takara, Japan), following the manufacturer’s instructions. RT-qPCR was performed using SYBR Green Real-time PCR Master Mix (Takara, Japan) with the Step One System (Life Technologies). The results were expressed as fold change from the untreated control and analyzed using the 2^−ΔΔCt^ method. The primers were as follows (5′–3′): MyD88 forward: TTT​ATC​TGC​TAC​TGC​CCC​AAC​G, reverse: GCG​GCG​ACA​CCT​TTT​CTC​A; TLR4 forward: ATG​CTG​CAA​CTG​ATG​TTC​CTT​C, reverse: GAT​GTT​AGA​CCT​TTC​TTC​CTC​CC; GAPDH forward: TGT​TCC​TAC​CCC​CAA​TGT​GTC​C, reverse: GGA​GTT​GCT​GTT​GAA​GTC​GCA​G; TNF-α forward: ATG​GCC​TCC​CTC​TCA​TCA​GT, reverse: TGG​TTT​GCT​ACG​ACG​TGG​G; IL-6 forward: AGT​GGC​TAA​GGA​CCA​AGA​C, reverse: ATA​ACG​CAC​TAG​GTT​TGC​CGA; iNOS forward: ATT​CAC​AGC​TCA​TCC​GGT​ACG, reverse: GGA​TCT​TGA​CCA​TCA​GCT​TGC; IL-1β forward: GCA​CTA​CAG​GCT​CCG​AGA​TGA​A, reverse: GTC​GTT​GCT​TGG​TTC​TCC​TTG​T.

### Isolation of immune cells

After the mice were anesthetized, 0.9% saline was used for transcardial perfusion. The right brain hemispheres were homogenized in a 6-well plate using 2 ml Hank’s balanced salt solution (HBSS, Solarbio) per well. Collagenase IV (1 mg/ml) was added to remove myelin. The brain homogenate was filtered through a 70 μm cell strainer and centrifuged at 300 *g* for 5 min at 4°C. Then, 2 ml of 30% Percoll (Sigma) were added to resuspend the brain cell precipitate, and the resuspended cells were slowly added to a 15 ml centrifuge tube containing 3 ml of 70% Percoll. The intermediate layer cells were analyzed by flow cytometry after density-gradient centrifugation.

### Flow cytometry

Approximately 5 × 10^5^ cells were suspended in 200 μL HBSS, and anti-mouse CD16/CD32 (5 ng/μL) was used to block Fc receptor binding. The cells were stained with allophycocyanin-conjugated CD45 antibody (1 ng/μL), FITC-conjugated CD11b antibody (1 ng/μL), and phycoerythrin-conjugated Ly6G antibody (1 ng/μL) and incubated in the dark at 4°C for 30 min. Finally, the cells were washed with HBSS buffer, resuspended in 200 μL of HBSS, and analyzed using a flow cytometer (BD FACSCalibur).

### Enzyme-linked immunosorbent assay

Enzyme-linked immunosorbent assay (ELISA) kits were used to detect the levels of TNF-α, IL-1β, and IL-6 in the brain. Brain tissue was homogenized in 1 ml of phosphate-buffered saline using a tissue homogenizer. Then, the homogenates were centrifuged at 14,000 *g* for 10 min at 4 °C. The supernatant was immediately transferred for measurement following the manufacturer’s instructions. BV-2 cells were incubated with different treatments and the supernatants were collected and measured.

### Capillary electrophoresis immunoassay (Simple Western)

Here, Simple Western, a novel immunoassay to detect proteins in the brain, was used, as previously described (Kannan et al., 2018; Nanki et al., 2018). Briefly, the prepared proteins were diluted to a concentration of 0.5 mg/ml using a sample preparation kit (Protein Simple, United States). Then, according to the manufacturer’s instructions, the prepared reagents were added to the detection plate sequentially for processing in an automated capillary electrophoresis system (Simple Western system). The primary antibodies recognized HMGB1, TLR4, MyD88, NeuN, P-ERK, P-IκBα, IκBα, NF-κB p65 (CST, 1:50), Histone H3 (ABclonal, 1:50), and GAPDH (ABclonal, 1:1000). Compass for SW software v4.0.0 (Protein Simple, United States) was used to quantitatively analyze the signal intensity (area) of the protein.

### Detection of drug concentration by liquid chromatography-mass spectrometry

The mice were intravenously injected with 15 mg/kg TJ-5 at the time of reperfusion 1 h after MCAO or Sham. The mice were then anesthetized at 5 min, 10 min, 30 min, 1 h, and 2 h, or 6 h post-dosing. Blood was collected to prepare serum samples, and the ipsilateral ischemic hemisphere (IL brain), contralateral non-ischemic hemisphere (CL brain), heart, and liver samples were obtained after transcardial perfusion with 0.9% saline. Liquid chromatography-mass spectrometry (LC-MS) was performed to determine the concentration of TJ-5 in the samples. The time-concentration curve, area under the curve (AUC), and pharmacokinetic (PK) parameters of TJ-5 were analyzed using the PKslover 2.0 PK software (Zhang et al., 2010). BBB permeability was evaluated using the brain-to-serum partition coefficient (K_p_), which was calculated as AUC_brain_/AUC_serum_.

### Statistical analysis

All experimental data were statistically analyzed using the professional analysis software GraphPad 8.0. The data obtained are expressed as mean ± standard error of the mean values (SEM). Different groups were compared with a *t*-test or two-way ANOVA, as appropriate. *p* < 0.05 was considered statistically significant.

## Results

### TJ-5 improves neurological function and reduces the infarct volume and neuronal loss in cerebral ischemia-reperfusion injury mice

To investigate the therapeutic effects of TJ-5 in cerebral I/R-induced acute injury, TJ-5 or edaravone was injected intravenously in the intervention groups at 1 h of ischemia, and CIRI mice were evaluated after 24 h of reperfusion (Figure 1B). The results showed that the neurological deficit score of the TJ-5 15 mg/kg group was significantly lower than that of the I/R group (Figure 1C). The infarct volume was evaluated using TTC staining. We found that TJ-5 significantly reduced the infarct volume, especially in the cortex, with an increase in dosage (Figure 1D). The percentage of infarct volume was 34.24% in the I/R group, while that in the most effective TJ-5 15 mg/kg group was only 8.47%, a reduction of approximately 80%. Furthermore, compared with 18.59% in the edaravone group, TJ-5 at 15 mg/kg achieved a better effect (Figure 1E). Intravenous TJ-5 at 4 h reperfusion still effectively reduced infarct volume (Supplemental Figure S1). We used TJ-5 at 15 mg/kg in the I/R + TJ-5 group in subsequent trials because of its superior effect. Immunofluorescence staining of neuron-specific nuclear protein (NeuN) in the brain showed that the number of neurons in the injured hemisphere in the I/R + TJ-5 group significantly increased compared to that in the vehicle group (Figure 1G). TJ-5 attenuated this decrease in NeuN protein levels (Figure 1H). Taken together, these data indicate that TJ-5 has powerful neuroprotective effects and reduces neuronal loss caused by CIRI.

### TJ-5 inhibits apoptosis and alleviates the excessive inflammatory response in CIRI mice

**FIGURE 2:**
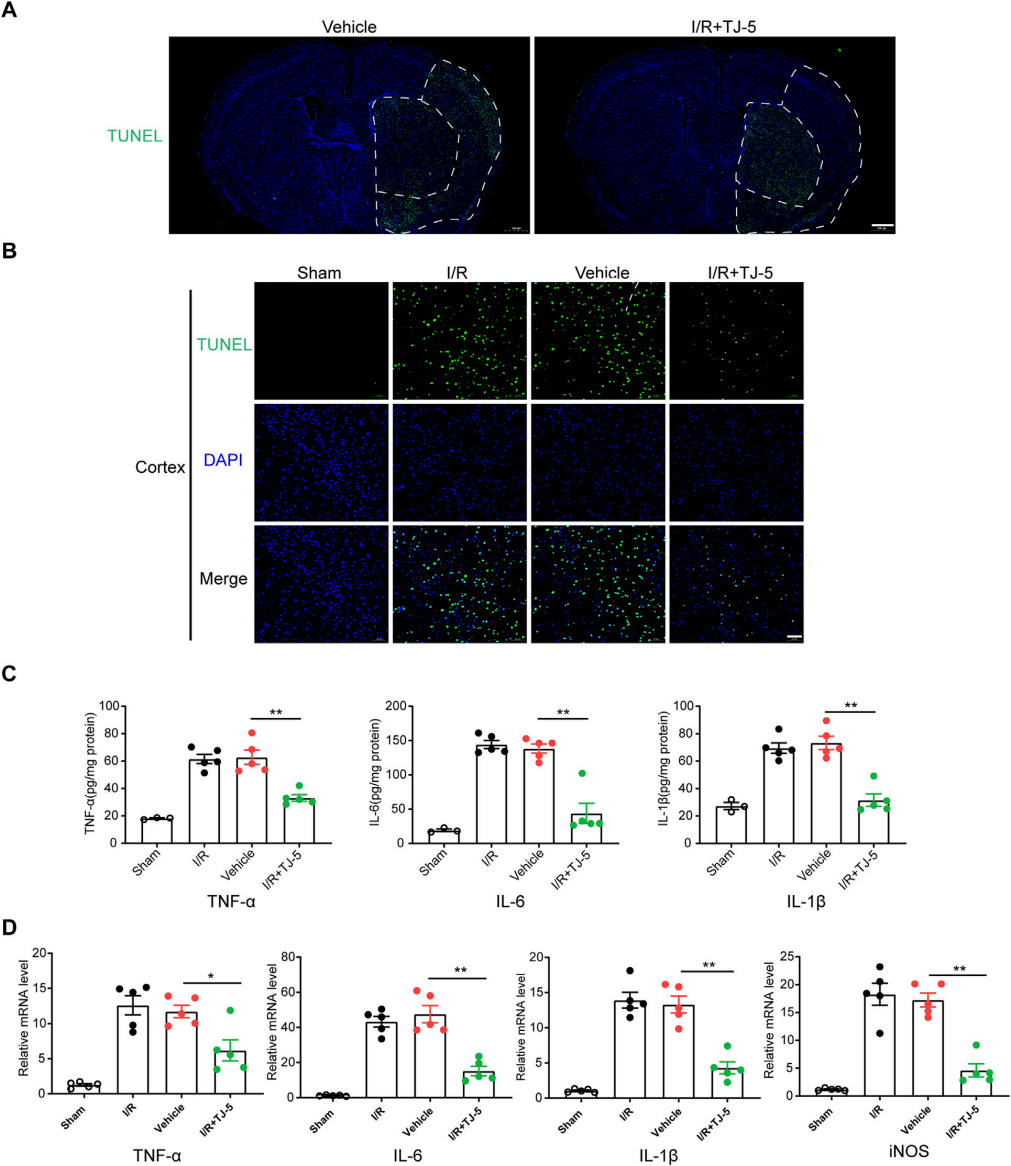
Effect of TJ-5 treatment on the neuroinflammatory response and apoptosis in CIRI mice. Mice were injected intravenously with TJ-5 (15 mg/kg) after 1 h ischemia. **(A)** Representative TUNEL staining image of whole brain slices (scale bar = 500 μm), white dashed line areas illustrate ischemic core (medial) and peri lesion (lateral) regions; the range of apoptosis in the brain was reduced with TJ-5. **(B)** Representative TUNEL staining image of cortex; the number of apoptotic cells were reduced with TJ-5 treatment (scale bar = 50 μm). **(C)** TNF-α, IL-6, IL-1β, and iNOS mRNA expression levels in the brain were downregulated with TJ-5 treatment. **(D)** The content of TNF-α, IL-6, and IL-1β in the brain were reduced with TJ-5 treatment. All the experiments were repeated three times. Values are mean ± SEM. (*p < 0.05, **p < 0.01).

To investigate the protective effects of TJ-5 on CIRI-induced inflammatory responses, TUNEL fluorescence staining was used to detect apoptosis. The scope of apoptosis in the whole brain and the apoptotic cells in the ischemic penumbra of the cortex were significantly reduced by treatment with TJ-5 (Figures 2A, B). The mRNA expression and protein levels of TNF-α, IL-1β, and IL-6 in the brain were detected by RT-qPCR and ELISA, respectively. The results showed that I/R injury significantly increased the expression and production of inflammatory factors in the brain tissue, whereas TJ-5 reduced the expression of TNF-α, IL-1β, and IL-6 (Figures 2C, D). These results suggested that TJ-5 reduced the extent of damage by inhibiting the neuroinflammatory response and apoptosis.

### TJ-5 inhibits activation of microglia and infiltration of peripheral myeloid cells in CIRI mice

**FIGURE 3:**
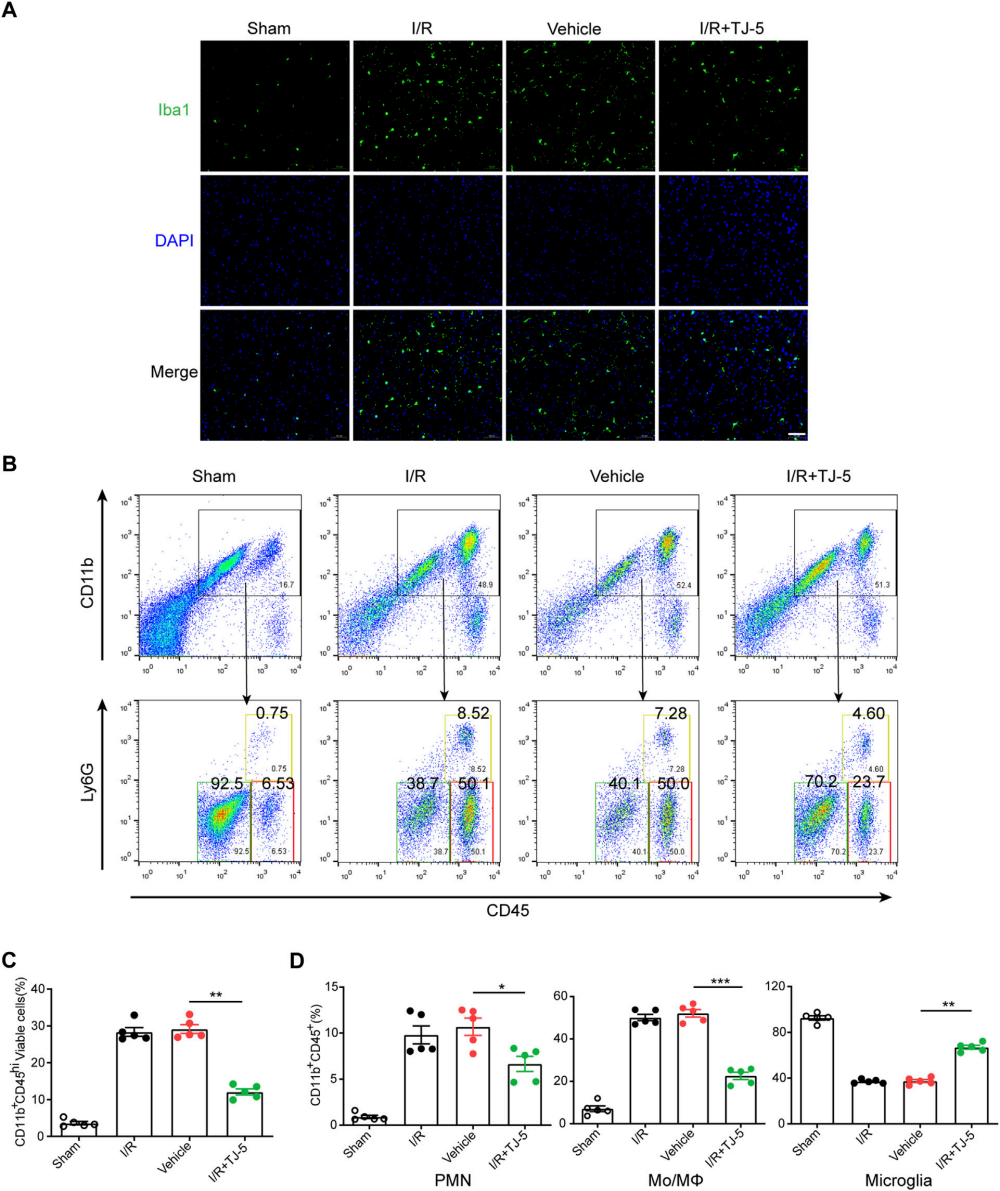
Effect of TJ-5 treatment on microglia and peripheral infiltrating myeloid cells in CIRI mice. Mice were injected intravenously with TJ-5 (15 mg/kg) after 1 h ischemia. **(A)** Representative Iba-1 staining image of the cortex (scale bar = 50 μm); the activated microglia were decreased with TJ-5 treatment. **(B)** Immune cells were isolated from the ischemic brain hemisphere after 24 h reperfusion and were stained with CD45, CD11b, and Ly6G. Plots identify CD11b^+^CD45^hi^Ly6G^−^ Mo/MΦ, CD11b^+^CD45^int^ microglia and CD11b^+^CD45^hi^Ly6G^+^ PMNs. **(C)** Percentage of brain-infiltrating CD11b^+^CD45^hi^ myeloid cells was statistically analyzed. **(D)** Percentages of PMNs, Mo/MΦ, and microglia in CD11b^+^CD45^+^ cells were statistically analyzed. All the experiments were repeated three times. Values are mean ± SEM. (*p < 0.05, **p < 0.01, ***p < 0.001).

We further explored how TJ-5 inhibited excessive inflammatory responses at the cellular level. Microglia in the brain were labeled with Iba-1, and the results indicated that the number of Iba-1 positive cells in the I/R group increased, while that in the I/R + TJ-5 group significantly decreased (Figure 3A). Flow cytometry results showed that the proportions of CD11b^+^CD45^hi^Ly6G^+^ neutrophils (PMNs) and CD11b^+^CD45^hi^Ly6G^−^ mononuclear macrophages (Mo/MΦ) increased, and the proportion of CD11b^+^CD45^int^ inactive microglia decreased in CIRI mice. TJ-5 attenuated this proportional change and inhibited the activation and infiltration of inflammatory cells (Figure 3B). Injecting TJ-5 at the time of reperfusion reduced the number of brain-infiltrating CD11b^+^CD45^hi^ myeloid cells and increased the proportion of inactive microglia (Figures 3C, D). Thus, TJ-5 inhibited both the infiltration of myeloid cells and activation of microglia to alleviate neuroinflammation in CIRI mice.

### TJ-5 inhibits neuroinflammation *via* the MyD88/NF-κB and ERK signaling pathway in CIRI mice

**FIGURE 4:**
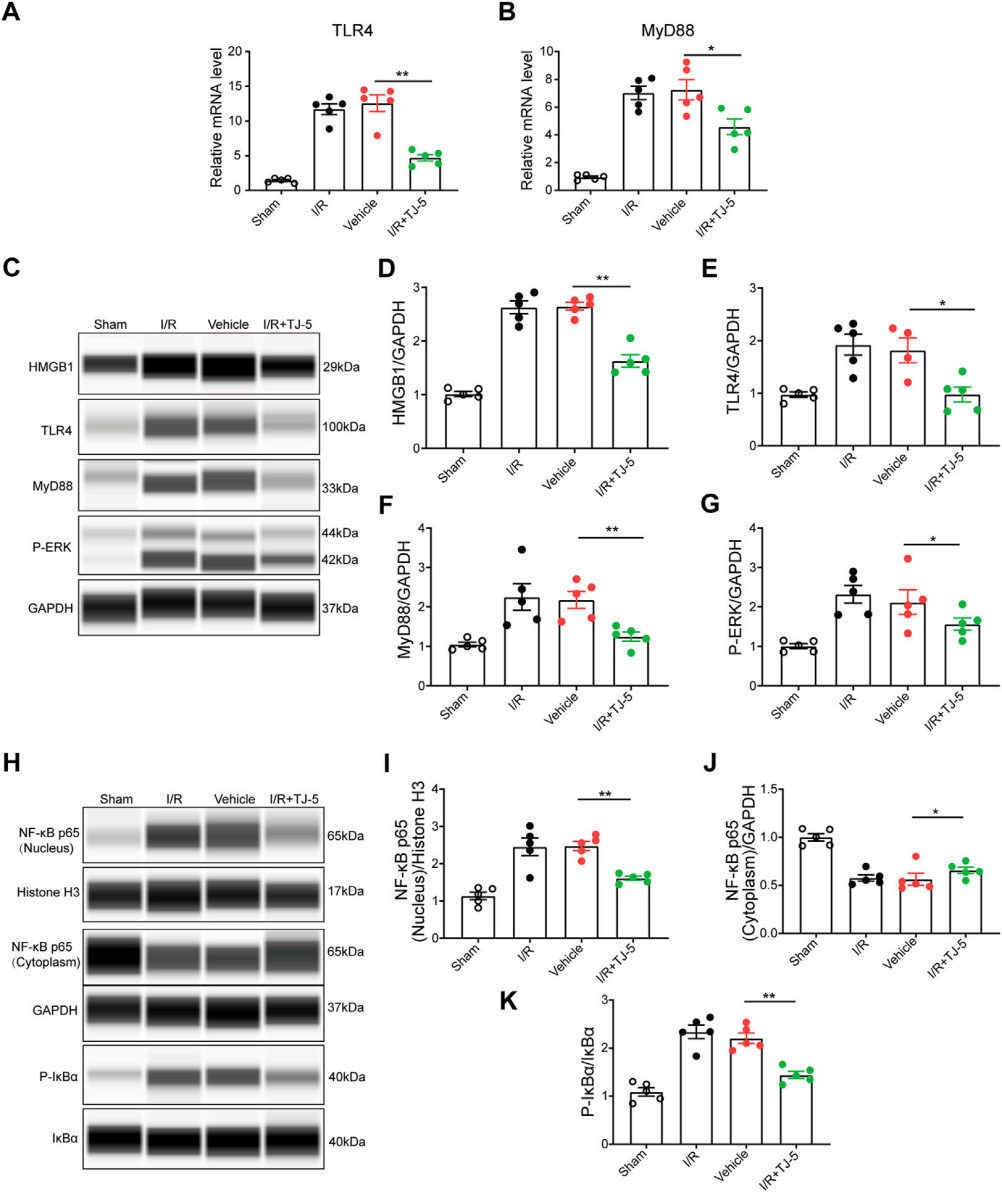
TJ-5 downregulates the MyD88/NF-κB and ERK signaling pathway in CIRI mice. **(A,B)** The mRNA levels of TLR4 and MyD88 in the brain were detected. **(C–G)** HMGB1, TLR4, MyD88, and P-ERK protein levels in the brain were assessed and analyzed. **(H–K)** Cytoplasmic and nuclear proteins were extracted from the brain to detect the NF-κB p65 protein levels and analyze phosphorylation of IκBα and NF-κB p65 nuclear translocation. All the experiments were repeated three times. Values are mean ± SEM. (*p < 0.05, **p < 0.01).

MyD88/NF-κB and ERK signaling plays a vital role in neuroinflammation induced by CIRI (Qin et al., 2022), and the effect of TJ-5 on this pathway in CIRI mice needs to be identified. The results showed that TJ-5 downregulated the expression of HMGB1, TLR4, and MyD88 and inhibited the phosphorylation of ERK in the ischemic hemisphere (Figures 4A–G). Phosphorylation of inhibitor complex alpha (IκBα) and nuclear translocation of NF-κB p65, which lead to the excessive expression of pro-inflammatory factors, were examined. The results indicated that phosphorylation of IκBα and nuclear translocation of NF-κB p65 was reduced by TJ-5 treatment in the ischemic hemisphere (Figures 4H–K). Therefore, TJ-5 inhibits the MyD88/NF-κB and ERK signaling pathway to alleviate neuroinflammation.

### Blood-brain barrier permeability and pharmacokinetics of TJ-5

**FIGURE 5:**
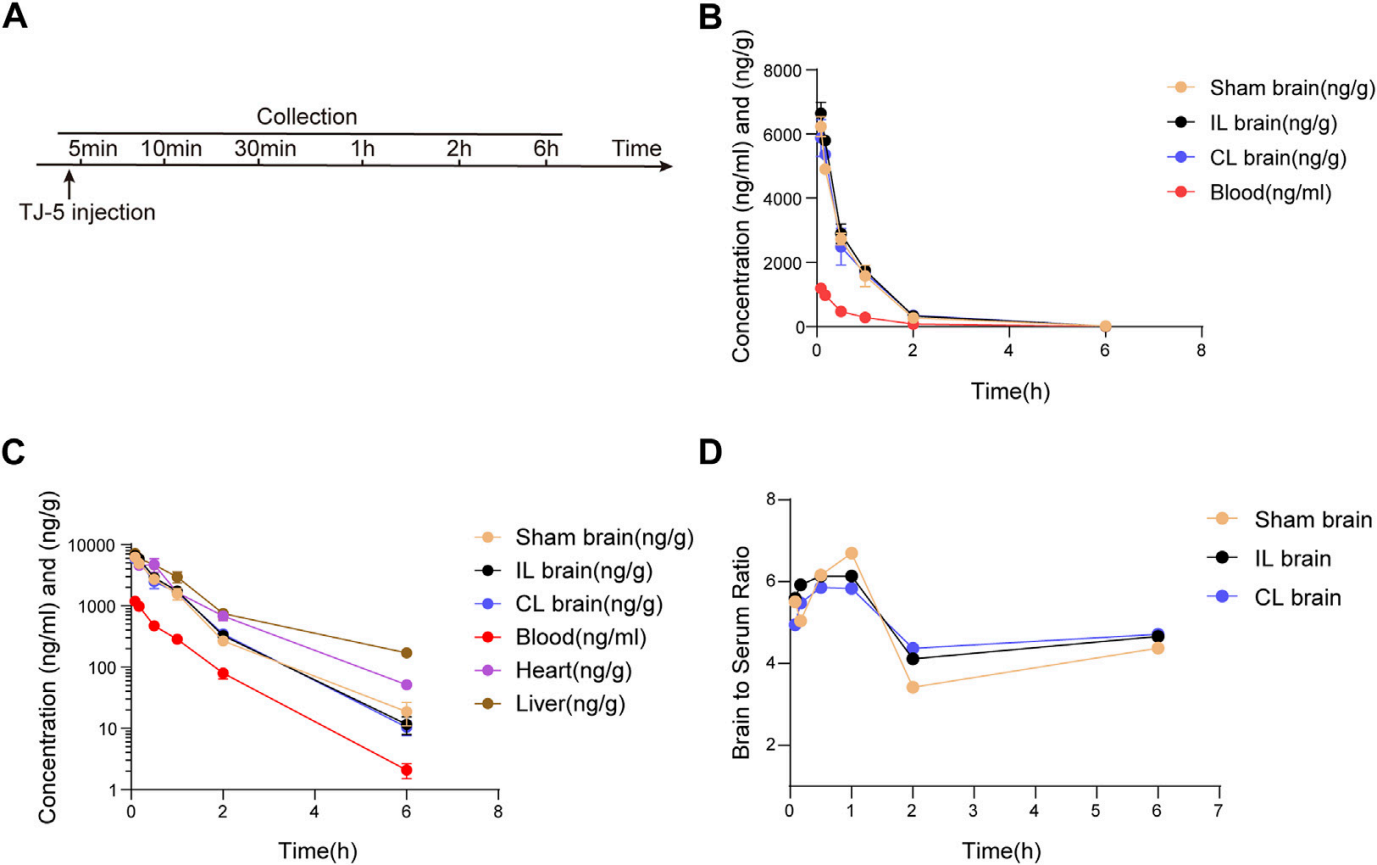
Pharmacokinetic profile of TJ-5. The concentrations of TJ-5 were analyzed in the serum, Sham brain, IL brain, CL brain, liver, and heart at specified time points after intravenous injection of TJ-5 at 15 mg/kg. **(A)** Experimental timeline of injection and detection. **(B)** The concentration-time profiles of TJ-5 in the serum and brain. TJ-5 was quickly distributed into the brain after intravenous injection. **(C)** The concentration-time profiles of TJ-5 with expressed in logarithmic ordinate. Elimination of TJ-5 followed the first-order elimination kinetics. **(D)** Concentration ratios of the brain to serum at 5 min, 10 min, 30 min, 1 h, 2 h, and 6 h. IL brain, ipsilateral ischemic hemisphere. CL brain, contralateral non-ischemic hemisphere.

To investigate the blood-brain barrier permeability of TJ-5, the concentration of TJ-5 in the IL and CL brains was measured and compared with that in the serum, liver, and heart (Figure 5A). The concentrations of TJ-5 in each tissue are summarized in Table 1 and the calculated PK parameters are summarized in Table 2. The time-concentration curves of TJ-5 in different tissues indicated that TJ-5 was eliminated according to first-order kinetics and that TJ-5 was rapidly distributed from the blood to the brain, heart, and liver after intravenous injection (Figures 5B, C). The TJ-5 concentration ratio of brain tissue to serum at each time point is shown (Figure 5D). It is well known that intense neuroinflammation occurring during the acute phase of cerebral ischemia is associated with BBB breakdown (Candelario-Jalil et al., 2022). Interestingly, the concentrations of TJ-5 in the Sham, IL and CL brains were similar, suggesting that the BBB may have little effect on the diffusion of TJ-5 into the brain. Thus, TJ-5 has good BBB permeability and can be quickly distributed in parenchymal organs with a short half-life.

**TABLE 1:** Distribution of TJ-5 in tissues during the different time points (ng/ml or ng/g, mean ± SD).

Sample	5 min	10 min	30 min	1 h	2 h	6 h
Serum	1187.80 ± 208.63	979.85 ± 202.81	471.74 ± 151.14	284.90 ± 29.10	79.60 ± 26.93	2.08 ± 1.12
Sham brain	6232.40 ± 435.71	4902.53 ± 53.92	2725.04 ± 256.34	1575.86 ± 468.76	267.68 ± 6.25	18.74 ± 11.04
IL brain	6646.07 ± 590.77	5798.80 ± 29.80	2893.70 ± 611.00	1747.40 ± 163.95	327.29 ± 57.19	11.65 ± 6.43
CL brain	5877.53 ± 989.32	5364.30 ± 749.83	2480.87 ± 985.41	1662.40 ± 117.03	347.65 ± 55.25	10.43 ± 5.69
Heart	5914.10 ± 824.68	4591.93 ± 351.85	4729.27 ± 2061.45	1655.33 ± 335.19	684.13 ± 198.89	51.60 ± 11.87
Liver	7162.10 ± 593.97	5890.47 ± 528.302	4713.00 ± 1130.68	2961.40 ± 1031.41	750.37 ± 249.40	169.90 ± 36.06

**TABLE 2:** PK parameters of TJ-5 in tissues of mice (mean ± SD).

Sample	Pharmacokinetic parameters	
Serum	AUC_0–6 h_ (ng/ml*h)	919.3 ± 82.24
	t_1/2_(h)	0.38
Sham brain	AUC_0–6 h_ (ng/ml*h)^a^	4579 ± 275.7
	t_1/2_(h)	0.65
	K_p_	4.98
IL brain	AUC_0–6 h_ (ng/ml*h)^a^	5136 ± 239.3
	t_1/2_(h)	0.41
	K_p_	5.58
CL brain	AUC_0–6 h_ (ng/ml*h)^a^	4792 ± 352.8
	t_1/2_(h)	0.41
	K_p_	5.21
Heart	AUC_0–6 h_ (ng/ml*h)^a^	6485 ± 768.9
	t_1/2_(h)	0.67
Liver	AUC_0–6 h_ (ng/ml*h)^a^	8238 ± 852.1
	t_1/2_(h)	0.70

^a^ The tissue density was assumed to be 1 g/ml. K_p_: Brain-serum ratio was calculated by the mean of AUC_0–6 h_ ratios. *t*_*1/2*_: elimination half-life.

### TJ-5 inhibits the activation of LPS- or OGD/R-stimulated BV-2 cells and apoptosis of OGD/R-induced SH-SY5Y cells

**FIGURE 6:**
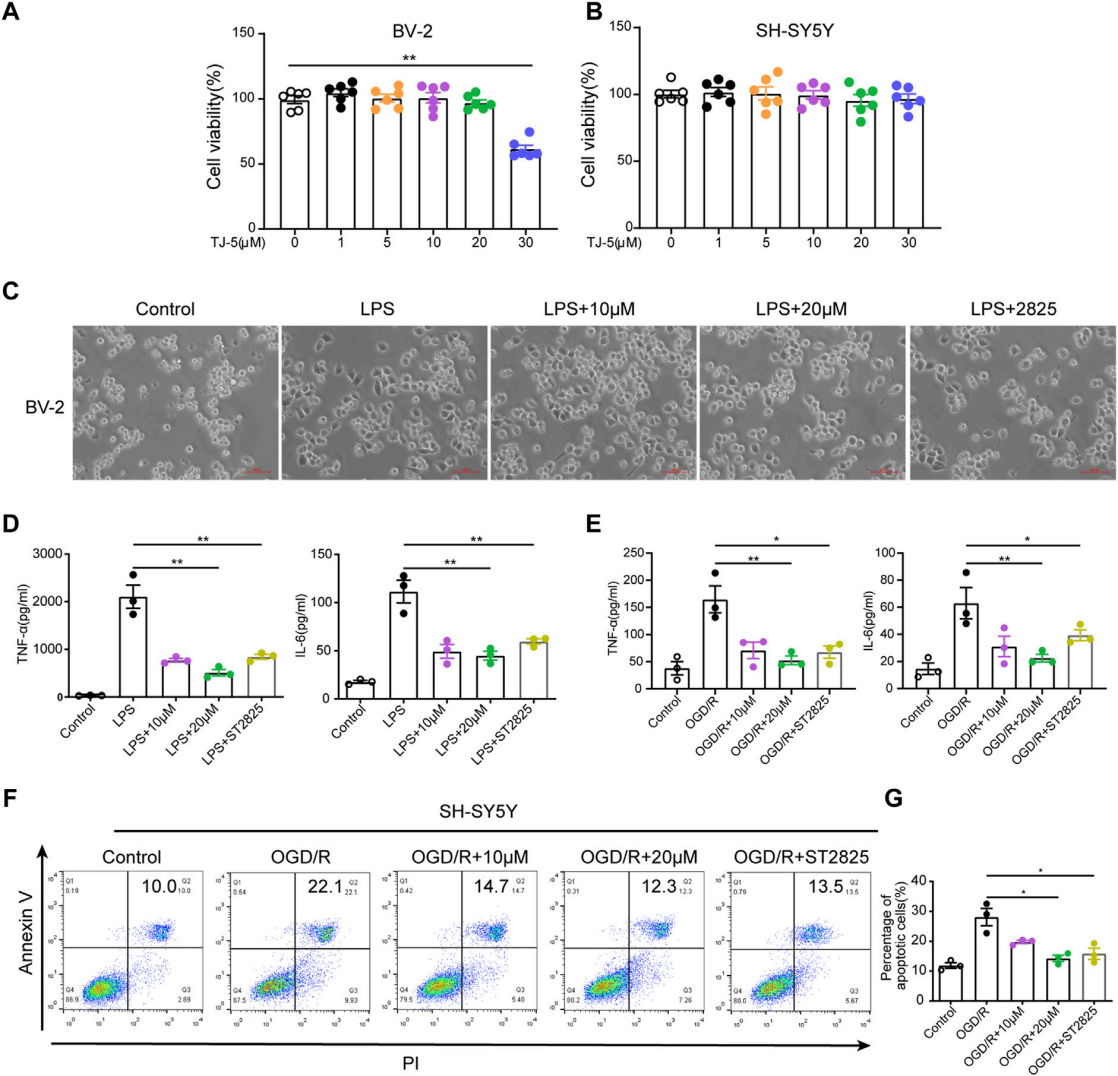
Effect of TJ-5 intervention on LPS or OGD/R-stimulated BV-2 cells and OGD/R-induced SH-SY5Y cells. BV-2 cells were pretreated with TJ-5 for 2 h before LPS or OGD/R stimulation for 24 h. SH-SY5Y cells were exposed to OGD for 4 h and then treated with TJ-5 during reperfusion for 24 h MyD88 inhibitor ST2825 was used as positive drug. **(A)** Cell viability of BV-2 cells at 24 h after TJ-5 intervention. **(B)** Cell viability of SH-SY5Y cells at 24 h after TJ-5 intervention. **(C)** The morphology of BV-2 cells was observed under the microscope. TJ-5 inhibited the activation of LPS-stimulated BV-2 cells (original magnification ×200). **(D)** TNF-α and IL-6 secretion in LPS-stimulated BV-2 cells was inhibited by TJ-5 intervention. **(E)** TNF-α and IL-6 secretion in OGD/R-stimulated BV-2 cells was inhibited by TJ-5 intervention. **(F,G)** Apoptosis of OGD/R-induced SH-SY5Y cells analyzed by flow cytometry. All the experiments were repeated three times. Values are mean ± SEM. (*p < 0.05, **p < 0.01).

The effects of TJ-5 on microglia and neurons were also evaluated *in vitro*. The CCK-8 experiment confirmed that TJ-5 did not affect the viability of BV-2 and SH-SY5Y cells at concentrations below 20 μM (Figures 6A, B). Observation of cell morphology under the microscope showed that after 24 h of LPS stimulation, BV-2 cells became amoeba-like and showed more protrusions, whereas the cells in the control group were spherical with a small number of protrusions. The activation of BV-2 cells was inhibited by TJ-5 intervention (Figure 6C). The levels of TNF-α and IL-6 in the supernatants of LPS- or OGD/R-stimulated BV-2 cells were markedly reduced by TJ-5 treatment (Figures 6D, E). In addition, an Annexin V/propidium iodide flow cytometry assay indicated that TJ-5 significantly reduced apoptosis of OGD/R-induced SH-SY5Y cells (Figures 6F, G). Meanwhile, ST2825 has the same anti-inflammatory effect. These results suggest that TJ-5 has anti-neuroinflammatory and neuroprotective effects on microglia and neurons, respectively.

### TJ-5 inhibits NF-κB p65 protein nuclear translocation in BV-2 cells

**FIGURE 7:**
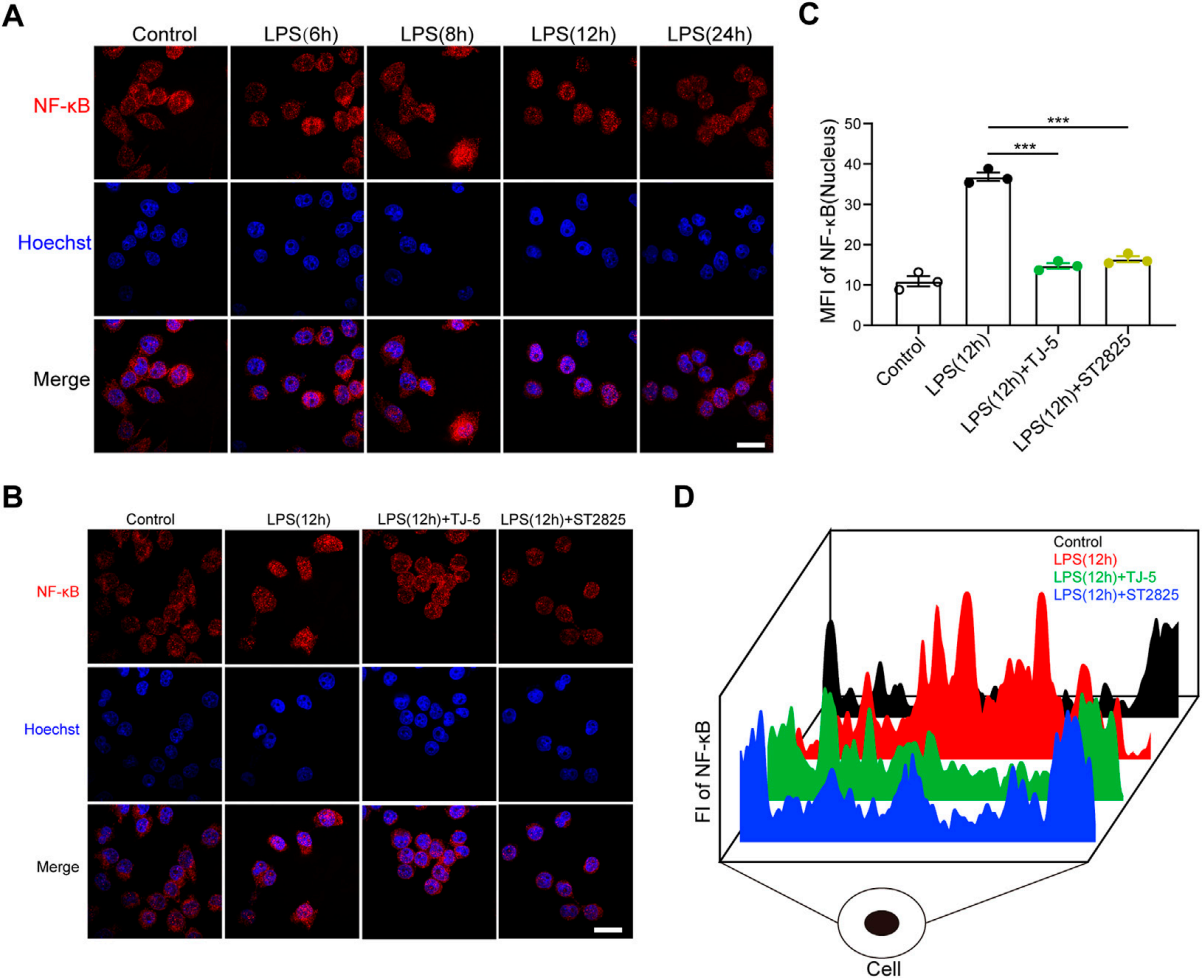
**(A)** Immunofluorescence was used to determine the optimal observation time point for NF-κB p65 nuclear translocation in LPS-stimulated BV-2 cells (scale bar = 50 μm). **(B)** After 12 h of LPS stimulation, immunofluorescence was used to analyze the effect of TJ-5 on NF-κB p65 nuclear translocation in BV-2 cells (scale bar = 50 μm). **(C)** The mean fluorescence intensity (MFI) of NF-κB p65 in the nucleus was statistically analyzed. **(D)** The distribution diagram of NF-κB p65 in cells was obtained by detecting fluorescence intensity (FI). All the experiments were repeated three times. Values are mean ± SEM. (***p < 0.001).

To further explore the effect of TJ-5 on the nuclear translocation of NF-κB p65 protein in microglia, NF-κB p65 protein in BV-2 cells was estimated by immunofluorescence. First, p65 content in the nucleus was observed at 6, 8, 12, and 24 h after LPS treatment. The results showed that p65 nuclear translocation was most obviofter 12 h of stimulation (Figure 7A). Subsequently, 12 h after stimulation was selected as the observation time point. The results showed that TJ-5 significantly prevented LPS-induced p65 nuclear translocation, which is same as ST2825 (Figures 7B–D). Therefore, the main mechanism by which TJ-5 inhibits excessive microglial activation may be the inhibition of NF-κB p65 nuclear translocation.

## Discussion

TJ-5 is a novel MyD88 pharmacological inhibitor. In this study, we evaluated the neuroprotective effects of TJ-5 in both *in vitro* and *in vivo* models of cerebral ischemia-reperfusion injury, explored its underlying mechanisms and investigated the druggability of inhibition of MyD88 in the brain.

**FIGURE 8:**
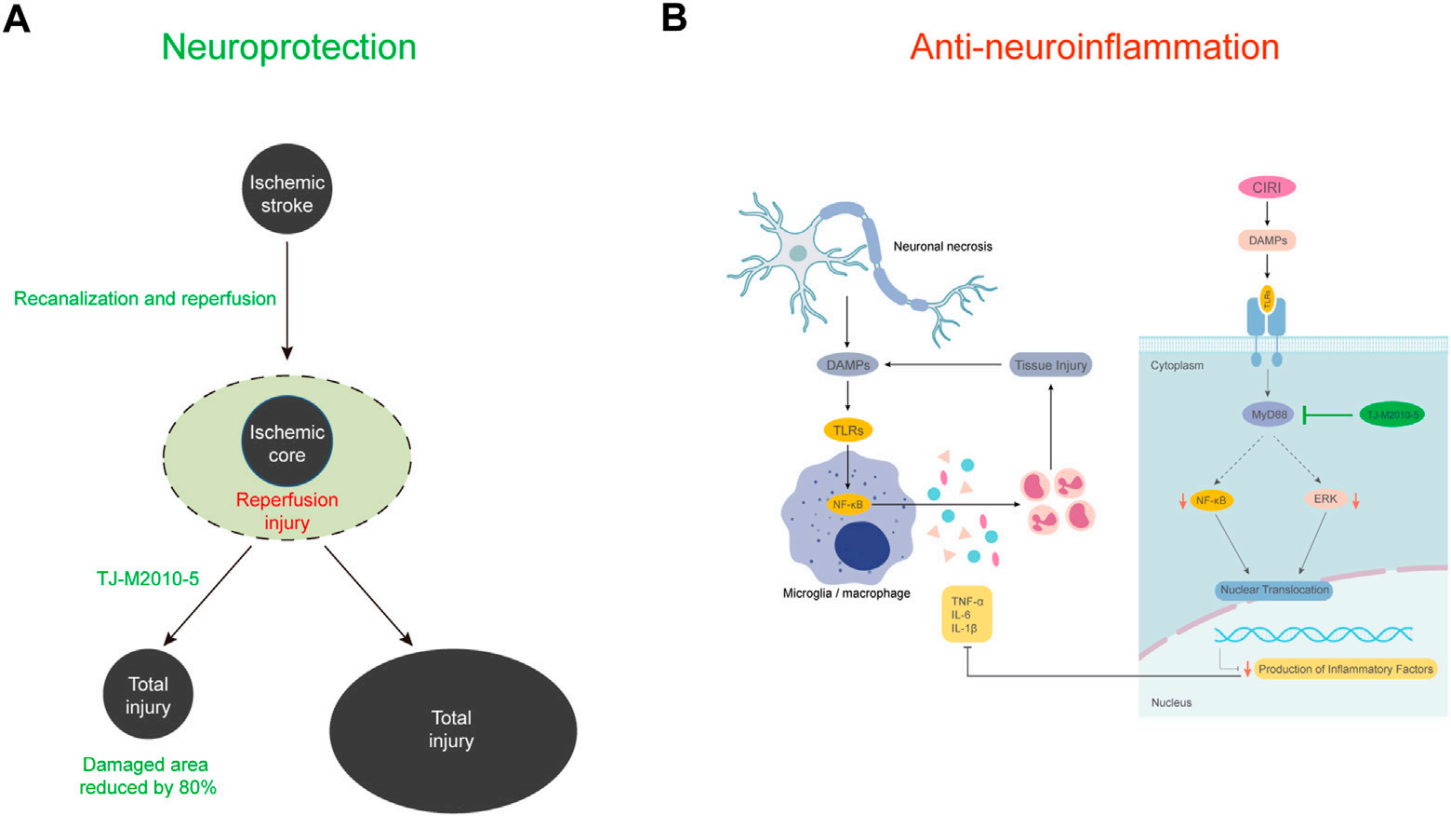
Schematic model of the present study. **(A)** The occurrence of reperfusion injury after vascular recanalization in ischemic stroke significantly expands the final necrotic area. TJ-5 reduces the final necrotic area by reversing ischemia-reperfusion injury. **(B)** The activation of microglia and infiltration of myeloid cells promote neuroinflammation and brain tissue injury after acute cerebral I/R. TJ-5 obstructs the vicious circle of neuroinflammatory injury by inhibiting MyD88/NF-κB and ERK signaling pathway.

Ischemic stroke has become one of the most common causes of disability and death worldwide (Virani et al., 2021). Recanalization as soon as possible is the primary treatment after ischemic stroke, but the ensuing reperfusion injury aggravates the brain injury and expands the infarct size. Unfortunately, there is currently a lack of specific treatment options (Qin et al., 2022). The TLR/MyD88/NF-κB signaling pathway has been found to be involved in the development of neuroinflammation injury in CIRI (Mitsios et al., 2006; Chen et al., 2022). MyD88 is a core transduction protein involved in this signaling pathway (Kawai and Akira, 2010). Considering that MyD88 activation may enhance neuroinflammation caused by ischemic stroke, we hypothesized that TJ-5 might potently inhibit neuroinflammation to protect against CIRI (Figures 8A, B). In CIRI mice model, we compared the neuroprotective effects of edaravone, a medicine currently used clinically for the treatment of ischemic stroke (Li et al., 2021), and different concentrations of TJ-5. We found that TJ-5 at 15 mg/kg was more effective, as the infarction volume was reduced by approximately 80%, achieving better neuroprotective effects than edaravone. Considering 3–4 h reperfusion is a clinically relevant therapeutic window in case of stroke, we performed and found that intravenous TJ-5 at 4 h reperfusion remained effective in reducing infarct volume. Meanwhile, TJ-5 reduced apoptosis of OGD/R-induced SH-SY5Y cells with no neurotoxicity. Many studies have suggested that the inflammatory response of the innate immune cell such as Mo/MΦ, PMN and microglia is essential for CIRI (Petrovic-Djergovic et al., 2016; Liu et al., 2019). Our findings proved that TJ-5 reduced the infiltration ratio of peripheral myeloid cells in the cerebral infarction area, increased the proportion of inactive microglia, and decreased the expression levels of TNF-α, IL-1β, and IL-6 in the infarction areas after 24 h of reperfusion, suggesting that TJ-5 may interrupt the inflammatory cascade and inhibit excessive neuroinflammation. Furthermore, we found that TJ-5 caused a reduction in inflammation of LPS- or OGD/R-stimulated BV-2 cells. These results indicate that TJ-5 has the potential to treat ischemic stroke and other CNS diseases caused by neuroinflammation.

Mechanistically, we explored NF-κB and ERK signaling pathway which were relevant to TLR4/MyD88 signaling and ischemia stroke (Zhang et al., 2021). NF-κB p65 protein binds to IκB protein and is present in the cytoplasm. IκB kinases (IKKs) act immediately after TLR4/MyD88 signaling to phosphorylate IκB and NF-κB p65, which results in the degradation of IκB and nuclear translocation of NF-κB p65 (Karin and Ben-Neriah, 2000). Reducing NF-κB p65 activity is associated with reduction in infarct volume after MCAO (Liu et al., 2018). Our findings indicated that TJ-5 inhibited NF-κB p65 signaling in the brains of CIRI mice and nuclear translocation of NF-κB p65 in BV-2 cells. The inhibition of P-ERK could produce a potential neuroprotective effect in ischemic stroke (Zhang et al., 2021). Our data suggested TJ-5 treatment showed lower level of P-ERK when compared to vehicle treatment. HMGB1, one of the major ligands for TLR, are significantly elevated and closely associated with neuroinflammation in CIRI (Singh et al., 2016). We found that TJ-5 reduced the expression of HMGB1, suggesting that the neuroinflammatory injury was alleviated. The results indicate that TJ-5 exerts its anti-inflammatory effect through the MyD88/NF-κB and ERK signaling pathway.

The BBB is an interface that controls the exchange of substances between the CNS and blood, which makes it difficult to develop drugs (Banks, 2016). Currently, many drugs are unable to enter the CNS efficiently through the BBB, which limits the development of therapies for CNS diseases (Chiu et al., 2021; Liu et al., 2021). Microglia are resident macrophages in the brain. The activation of microglia in the CNS is an important factor contributing to the occurrence and development of CIRI (Cheng et al., 2019; Shen et al., 2022). In CIRI mice, activation of endogenous microglia and infiltration of exogenous immune cells promote a cascade of inflammation in the brain and increase the scope of injury. Inhibiting microglial activation is the focus of research on drugs for treating ischemic stroke (Ye et al., 2019; Wang et al., 2020). In CNS drug discovery, the BBB permeability of drugs is an essential factor, as it determines whether drugs can directly affect microglia in the brain. Therefore, the inhibitory effects of TJ-5 on microglial activation and BBB permeability were examined in the present study. Pharmacologic evidence demonstrates that TJ-5, a small-molecule compound, can pass through the BBB and directly inhibit the activation of microglia. Moreover, TJ-5 has a short half-life, can only maintain effective concentration for about 6 h with a single intravenous injection of near toxic dose, suggesting that continuous low-dose intravenous infusion should be considered. More PK data with the other dose regimens will be performed in future clinical trials. The simultaneous inhibition of peripheral myeloid cells and microglial activation may be responsible for the excellent efficacy of TJ-5 for abating the negative effect of CIRI. These results indicate that TJ-5 has significant clinical application value in the treatment of CIRI and suggest the druggability of inhibition of MyD88 in the brain.

This study affirms the potency of TJ-5 in treating CIRI, as it demonstrates a better neuroprotective effect in the early stage of cerebral I/R. Moreover, we verified that TJ-5 not only acts on peripheral innate immune cells, but also directly on cells in the brain, which may be an influential factor contributing to its exceptional anti-neuroinflammatory and neuroprotective effects. However, this study had some limitations. The inflammatory response to CIRI is a “double-edged sword”. An excessive inflammatory response causes the injury to expand, but the inflammatory response also promotes the immune cells to devour necrotic tissue, which can promote tissue repair (Wyss-Coray and Mucke, 2002; Xue et al., 2021). TJ-5 regulates the neuroinflammatory response in CIRI and is effective in the acute phase; however, its efficacy in the chronic phase requires further investigation. One study showed that a congenital deficit of MyD88 failed to reduce cerebral infarct size in MyD88 knockout mice, but MyD88-dependent signaling contributes to the inflammatory responses induced by cerebral I/R (Ye et al., 2016). Why there is the difference in efficacy between congenital defects and the acquired short-term inhibition of MyD88? As the downstream of MyD88, the different roles of NF-κB activity in the early and late stages of ischemic stroke may be an explanation (Ridder and Schwaninger, 2009). One report claims that the anti-apoptotic properties of NF-κB may indeed have an effect at late stage of transient cerebral ischemia (Duckworth et al., 2006). Nijboer et al. (2008) found that inhibition of early NF-κB-activity by intraperitoneal administration of the NF-κB inhibitor TAT-NBD at 0/3 h has strong neuroprotection in neonatal hypoxia-ischemia model, whereas inhibition of both early and late NF-κB-activity at 0/6/12 h or only late NF-κB activity at 18/21 h aggravated cerebral damage. They suggest that inhibition of early NF-κB activity is neuroprotective only when late NF-κB activity is maintained. Therefore, short-term inhibition of MyD88 attenuates cerebral damage in ischemic stroke. Considering the rapid diffusion of the drug and its short half-life, the selection of the intensity and duration of intervention with TJ-5 in this study has limitations. Taken together, for aseptic inflammatory reactions like CIRI, it can be concluded that the key to the treatment of CIRI is to balance the regulation of the immune system and minimize neuron loss. The anti-CIRI effect of TJ-5 should be evaluated in clinical studies.

## Conclusion

In summary, we confirmed that MyD88 inhibitor TJ-5 has an impressive therapeutic effect during the acute phase of CIRI as an emergency drug candidate by inhibiting neuroinflammation. Moreover, we clarified for the first time that the druggability of MyD88 in the CNS to TJ-5. We found TJ-5 can cross the BBB to directly inhibit the activation of microglia with no neurotoxicity. TJ-5 attenuates intense neuroinflammation *via* the MyD88/NF-κB and ERK signaling pathway.

## Data Availability

The original contributions presented in the study are included in the article/Supplementary Material, further inquiries can be directed to the corresponding authors.
