# Supplementary material for: TJ-M2010-5, a novel CNS drug candidate, attenuates acute cerebral ischemia-reperfusion injury through the MyD88/NF-κB and ERK pathway
Source: Front Pharmacol. 2022 Dec 15;13:1080438. doi: 10.3389/fphar.2022.1080438 (PMC9797592; doi:10.3389/fphar.2022.1080438)

Supplemental Figure I. Mice were injected intravenously with TJ-5 at 4 hours reperfusion. Representative TTC-stained slices at 24 h after reperfusion and statistical analysis of infarct volume. Values are mean ± SEM and analyzed by two-way ANOVA (**P < 0.01).

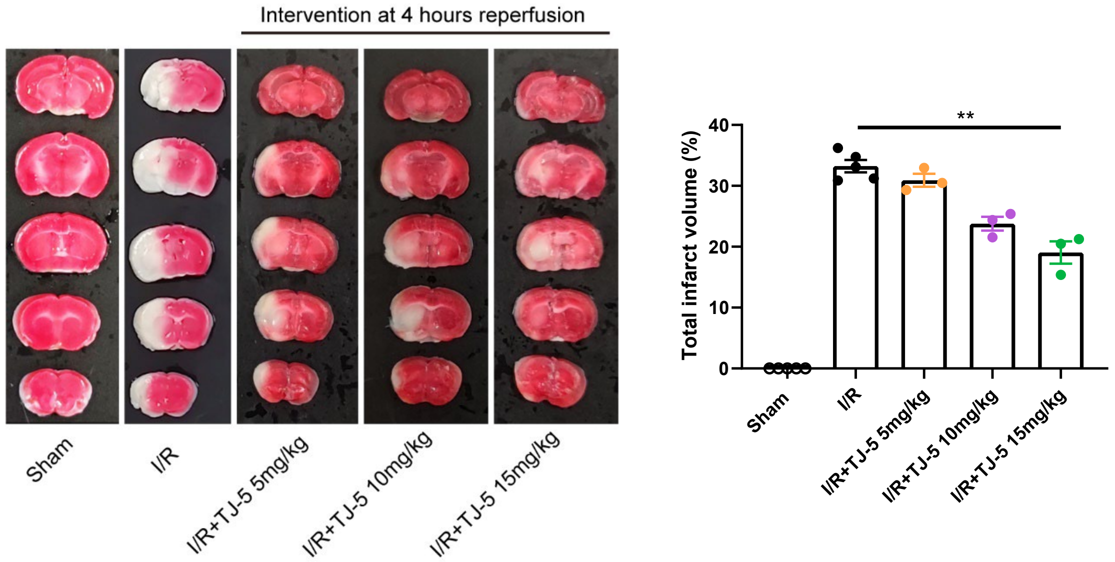

Supplement: Supplementary file 1 [file Image1.pdf]
